# Cerebral amyloid angiopathy is associated with decreased functional brain connectivity

**DOI:** 10.1016/j.nicl.2020.102546

**Published:** 2020-12-24

**Authors:** Nadieh Drenth, Jeroen van der Grond, Serge A.R.B. Rombouts, Mark A. van Buchem, Gisela M. Terwindt, Marieke J.H. Wermer, Jasmeer P. Chhatwal, M. Edip Gurol, Steven M. Greenberg, Sanneke van Rooden

**Affiliations:** a Department of Radiology, Leiden University Medical Center, P.O. Box 9600, 2300 RC Leiden, The Netherlands; b Leiden University, Institute of Psychology, P.O. Box 9555, 2300 RB Leiden, The Netherlands; c Leiden Institute for Brain and Cognition, P.O. Box 9600, 2300 RC Leiden, The Netherlands; d Department of Neurology, Leiden University Medical Center, P.O. Box 9600, 2300 RC Leiden, The Netherlands; e Department of Neurology, Massachusetts General Hospital, Harvard Medical School, 175 Cambridge Street, Suite 300, Boston, MA 02114, USA

**Keywords:** Functional connectivity, Resting state networks, Cerebral amyloid angiopathy, Functional magnetic resonance imaging, Neuroimaging

## Abstract

Cerebral amyloid angiopathy (CAA) is a major cause of intracerebral hemorrhage and neurological decline in the elderly. CAA results in focal brain lesions, but the influence on global brain functioning needs further investigation. Here we study functional brain connectivity in patients with Dutch type hereditary CAA using resting state functional MRI. Twenty-four DNA-proven Dutch CAA mutation carriers (11 presymptomatic, 13 symptomatic) and 29 age-matched control subjects were included. Using a set of standardized networks covering the entire cortex, we assessed both within- and between-network functional connectivity. We investigated group differences using general linear models corrected for age, sex and gray matter volume. First, all mutation carriers were contrasted against control subjects and subsequently presymptomatic- and symptomatic mutation carriers against control subjects separately, to assess in which stage of the disease differences could be found. All mutation carriers grouped together showed decreased connectivity in the medial and lateral visual networks, default mode network, executive control and bilateral frontoparietal networks. Symptomatic carriers showed diminished connectivity in all but one network, and between the left and right frontoparietal networks. Presymptomatic carriers also showed diminished connectivity, but only in the frontoparietal left network. In conclusion, global brain functioning is diminished in patients with CAA, predominantly in symptomatic CAA and can therefore be considered to be a late consequence of the disease.

## Highlights

• Functional connectivity is diminished across the cortex in Dutch-type CAA.
• Lower functional connectivity can already be found in presymptomatic CAA patients.
• Decreased functional connectivity is most pronounced in symptomatic CAA patients.

## 1 Introduction

Sporadic cerebral amyloid angiopathy (CAA) is an increasingly recognized cause of intracerebral hemorrhages (ICHs), loss of neurological function and cognitive decline. The disease is caused by an excessive deposition of amyloid β peptides in the small leptomeningeal and cortical vessels (Charidimou et al., 2017). CAA is a common finding among the elderly and often accompanies other brain pathologies, such as Alzheimer’s disease (AD) (Keage et al., 2009, Brenowitz et al., 2015). A large post-mortem study in almost 4000 elderly subjects, age of death ≥ 65 years, found evidence for CAA in up to 30% of non-demented, and up to 70–98% of demented subjects, depending on AD severity (Brenowitz et al., 2015). In total, 65% of all participants, both non-demented and demented, presented with some degree of CAA (30.5% mild CAA, 21.5% moderate CAA, and 13.7% severe CAA) (Brenowitz et al., 2015).

Even though a definitive diagnosis of CAA can only be made post-mortem, research has identified numerous neuroimaging biomarkers that can facilitate a reliable in vivo diagnosis of CAA. A standardized set of criteria have been developed based on these radiological findings (primarily the presence and location of intracerebral hemorrhages) with which a diagnosis of CAA can be made in vivo with a sensitivity of 94.7% and specificity of 81.2% (modified Boston criteria; Linn et al., 2010). Besides lobar intracerebral hemorrhages, other key neuroimaging markers of the disease include strictly lobar cerebral microbleeds (CMBs), posterior dominant white matter hyperintensities (WMHs), centrum semiovale enlarged perivascular spaces (CSO-EPVS), and cortical superficial siderosis (cSS) (Charidimou et al., 2017).

The mechanisms for CAA-related brain damage are still incompletely understood. It is crucial to gain more insight into CAA-related brain damage, considering the high prevalence of sporadic CAA in the population. Moreover, its comorbidity with AD increases its burden on society immensely. For a better understanding of disease mechanisms, CAA-related effects need to be disentangled. Thus, further insights into CAA are not only important for CAA itself, but can also advance the knowledge of AD pathology and development of AD treatments.

Recently, the association between brain connectivity and CAA has emerged as a promising new avenue of exploration. Structural brain connectivity is affected globally in CAA patients showing decreased strength and efficiency of structural networks which inversely correlates with lesion burden and disease progression over time (Reijmer et al., 2015, Reijmer et al., 2016, Schouten et al., 2019, Valenti et al., 2017). However, to date, no studies have investigated brain connectivity in CAA from a functional perspective, even though it has become increasingly recognized as a powerful method to objectively study underlying disease mechanisms, early signs of disease onset, and monitor disease progression of neurodegenerative disease (Zhou et al., 2017). Functional brain connectivity reflects the degree of synchronized activity between brain regions and can be assessed using functional magnetic resonance imaging (fMRI). Clustering of synchronized brain regions results in a highly reproducible set of the so called ‘Resting State Networks’ (RSNs) (Beckmann et al., 2005, Damoiseaux et al., 2006, Smith et al., 2009), where abnormal connectivity in the RSNs is characteristic of specific diseases and/or disease states (Zhou et al., 2017).

The aim of this study is to investigate the global impact of CAA on the brain by studying functional connectivity in patients with Dutch-type CAA (D-CAA, also referred to as hereditary cerebral hemorrhage with amyloidosis – Dutch type or HCHWA-D). D-CAA is a autosomal dominant form of CAA which is caused by a Glu693Gln mutation in the APP gene and has similar underlying pathology to sporadic CAA (Bornebroek et al., 1996, Maat-Schieman et al., 1996, Zhang-Nunes et al., 2006). Carriers of the D-CAA mutation can be identified by DNA testing, facilitating early identification and providing the unique opportunity to study the presymptomatic stage of the disease. Eventually, all mutation carriers will develop symptomatic CAA. D-CAA can be seen as a pure CAA model largely unaffected by confounding factors often found in sporadic CAA patients such as aging and other comorbidities (Bornebroek et al., 1996), as disease onset is up to 30 years earlier in life than in sporadic CAA (Zhang-Nunes et al., 2006). The main research question is if D-CAA mutation carriers show different functional connectivity patterns compared with age-matched control subjects. As CAA pathology preferentially affects the posterior brain regions, particularly the occipital cortex (Freeze et al., 2019, Kövari et al., 2013, Vinters and Gilbert, 1983), we expect to find differences in functional connectivity of RSNs encompassing the occipital cortex. Additionally, we explore if functional connectivity differences can already be seen in the presymptomatic stage of the disease, as this would be an important stage for early intervention opportunities. Lastly, we investigate the relationship between functional connectivity and CAA lesion burden (as indicated by the key neuroimaging markers). Similar to structural connectivity studies, we expect to find an inverse association between lesion burden and functional connectivity.

## 2 Methods

### 2.1 Study design and participants

This case-control study was performed at the Departments of Radiology and Neurology of the Leiden University Medical Center (LUMC; Leiden, the Netherlands) and has been previously described elsewhere (van Rooden et al., 2016). Participants were recruited through the HCHWA-D patient association (Katwijk, the Netherlands) and the outpatient clinic of the Department of Neurology of the LUMC, based on DNA analysis for confirmation of the Glu693Gln mutation in the APP gene. Additional participants were recruited from individuals at risk of HCHWA-D (i.e. a parent has HCHWA-D) and from participants’ spouses, family, or friends. All participants underwent genetic testing. Participants were included in the symptomatic group if they had a positive genetic test, had experienced signs of the disease reported to a general practitioner, and previously had one or more intracerebral hemorrhages. Participants were included in the presymptomatic group if they had a positive genetic test, but had not reported any signs of the disease to a general practitioner. Participants with a negative genetic test were included in the control group, which was age-matched to the patient groups. All control subjects were ascertained to be stroke-free. All participants underwent extensive neuropsychological and neuropsychiatric testing. Both presymptomatic mutation carriers and control subjects scored within normal range on all of the tests (previously published in van Rooden et al., 2016). In total, 27 mutation carriers (12 presymptomatic and 15 symptomatic) and 33 age-matched control subjects participated. The ethics committee of the Leiden University Medical Center approved the study and written informed consent was obtained from all participants.

For the present study, participants with both a structural MRI scan as well as a resting state fMRI scan were selected (*n* = 56). Three participants were subsequently excluded due to either excessive head movement (>3 mm movement in any direction; *n* = 1), incomplete field of view (FOV; *n* = 1), or failure to complete the resting state fMRI scan (*n* = 1). The remaining 53 participants were included of which there were 24 mutation carriers (11 presymptomatic and 13 symptomatic) and 29 control subjects.

### 2.2 MRI acquisition

All participants were scanned at the Leiden University Medical Center on a 3-Tesla Philips Achieva MRI scanner (Philips Medical Systems, Best, the Netherlands) using a standard 32-channel head coil. The scanning session included multiple sequences particularly suited for lesion detection (e.g. fluid-attenuated inversion recovery and susceptibility-weighted images, described in van Rooden et al., 2016), as well as a three-dimensional T1-weighted high-resolution structural image and a resting state functional MR image (RS-fMRI). Parameters for the structural image were: echo time (TE) 4.6 ms, repetition time (TR) 9 ms, flip angle 8°, 140 slices, FOV 224 × 177 × 168 mm, and scan duration 4 min 56 s. Resting state T2*-weighted images were acquired with echo planar imaging with TE 30 ms, TR 2200 ms, flip angle 80°, 38 slices, and FOV 220 × 220 × 115 mm, resulting in a voxel size of 2.75 × 2.75 × 3.03 mm, including a 10% interslice gap, 200 volumes, and scan duration 7 min 29 s. Two dummy scans preceded RS-fMRI acquisition for T1 stabilization purposes. Participants were instructed to lie as still as possible. For the RS-fMRI scan, participants were additionally instructed to keep their eyes closed and not to fall asleep.

### 2.3 Image processing

#### 2.3.1 Lesion burden

To assess the total lesion burden of each subject, a composite score of the ‘total MRI burden of SVD in CAA’ was computed, as defined by Charidimou et al. (2016). The composite score was comprised of four of the major imaging characteristics of the disease, that is lobar CMBs, WMHs, CSO-EPVSs and cSS. Each neuroimaging marker received a subscore of 0 or 1 for WMHs and CSO-EPVS, or between 0 and 2 for lobar CMBs and cSS, based on their validated rating systems. These subscores were then summed to create an ordinal scale ranging from 0 to 6, with higher scores indicating higher CAA lesion burden. For this purpose, deep and periventricular WMHs were assessed with the Fazekas visual rating scale and dichotomized into the WMHs subscore, where 1 point was assigned in case of confluent deep WMHs (Fazekas 2 or 3) and/or irregular periventricular WMHs extending into the deep white matter (Fazekas 3). If these criteria were not met, 0 points were awarded. CSO-EPVS was defined and scored according to a 4-point scoring system and dichotomized into the CSO-EPVS subscore. One point was awarded if rated moderate or severe (degree 3 or 4), otherwise 0 points were awarded (absent or degree 1 or 2). For lobar CMBs, a count of less than two received 0 points on the subscale, two to four lobar CMBs resulted in a subscore of 1, and five or more lobar CMBs in a subscore of 2. Lastly, cSS was scored according to a visual rating system, classifying cSS as either focal (restricted to ≤ 3 sulci) or disseminated (≥4 sulci). No cSS present resulted in a subscore of 0, focal cSS received a subscore of 1, and disseminated cSS received a subscore of 2.

In addition to determining the severity of WMHs with the Fazekas scoring system, we determined the volume of white matter hyperintensities. WMHs were segmented using a semi-automated method as described previously (Hafkemeijer et al., 2014). In short, for each subject white matter masks were created by segmentation of the 3DT1 image and spatially transformed to the subject’s FLAIR image. A threshold was applied to identify hyperintense white matter voxels on FLAIR. The resulting WMH maps were manually checked and edited for quality control and total volume of hyperintensities was calculated.

Gray matter (GM) volumes were calculated by segmenting subjects’ high-resolution structural images into GM, WM and CSF using FSL’s SIENAX (Smith et al., 2002, Smith et al., 2004). All GM partial volume maps were visually inspected to ensure no hemorrhagic tissue or WMHs were included. Misclassification of WMHs as GM were manually corrected by subtracting each subject’s WMH map from the subject’s GM partial volume map. Next, GM volumes were calculated and normalized for subject head size by multiplying with a subject specific scaling factor as calculated by SIENAX.

#### 2.3.2 Preprocessing

High-resolution structural and resting state fMRI scans were preprocessed and analyzed using FMRIB’s Software Library (FSL 5.0.11, www.fmrib.ox.ac.uk/fsl, Jenkinson, Beckmann et al., 2012). Before preprocessing, structural and resting state images were submitted to a visual quality check, to ensure that no major artifacts were present. Structural images were then skull stripped using the brain extraction tool of FSL (Smith, 2002). Preprocessing of the resting state images was performed using FEAT (FMRI Expert Analysis Tool) and included brain extraction (Smith, 2002), motion correction using MCFLIRT (Jenkinson et al., 2002), and spatial smoothing using a Gaussian kernel of FWHM 5 mm. Additionally, registration parameters were calculated using FLIRT (Jenkinson and Smith, 2001, Jenkinson et al., 2002) and FNIRT (Andersson et al., 2007). To calculate the registration parameters, functional images were registered to the subject’s high-resolution structural image using Boundary-Based Registration. Structural images were registered to the 2 mm isotropic MNI-152 standard space image (Montreal Neurological Institute, Montreal, QC, Canada) using non-linear registration with a warp resolution of 10 mm. Furthermore, we employed ICA-AROMA (Pruim et al., 2015a, Pruim et al., 2015b), an ICA-based strategy for Automatic Removal of Motion Artifacts, for secondary denoising. Next, high-pass temporal filtering with a cut-of frequency of 0.01 Hz was applied using FEAT. Lastly, functional images were warped to the 2 mm isotropic MNI standard space image using the registration parameters calculated before.

#### 2.3.3 Dual regression

To study functional connectivity, we used a dual regression approach with standardized network templates. Standardized RSN templates were chosen to improve generalizability and comparability to other study samples. Ten standard RSN templates (Smith et al., 2009) were used, comprising the visual medial network, visual occipital network, visual lateral network, default mode network, cerebellar network, sensorimotor network, auditory network, executive control network, frontoparietal right network and frontoparietal left network. The ten RSN templates, together with a CSF and a WM template for nuisance reduction, were entered into a dual regression. In the dual regression, the spatial maps of the twelve templates (ten networks, CSF and WM templates) were first regressed against each participant’s preprocessed resting state image, resulting in subject-specific time courses for each template. These time courses were then entered into a temporal regression against the same preprocessed resting state images to produce subject-specific spatial maps. In the resulting spatial maps, each voxel has a value (*z* score), which indicates the correlation of that voxel to the mean time course of a given template. Due to limited brain coverage of the cerebellum in most participants, the cerebellar network was excluded from further analyses. It was however decided to keep the network in the dual regression for nuisance reduction, to regress out signal belonging to the cerebellar network. Therefore, a total of nine RSNs were evaluated in the statistical analysis.

### 2.4 Statistical analysis

All statistical analyses were performed in SPSS (version 25.0). Demographics and characteristics were compared between control subjects and mutation carriers with independent *t*-tests for normally distributed variables and Mann-Whitney U tests for variables with a non-normal distribution. When comparing presymptomatic- and symptomatic mutation carriers with control subjects separately, ANOVAs with post hoc Bonferroni testing or Kruskal-Wallis tests with pairwise comparisons using Dunn’s procedure with Bonferroni correction were used. For categorical variables Chi-Square tests were used, except in cases where the sample size adequacy assumption was violated, then Fisher’s Exact tests were used instead. Significant Chi-Square or Fisher’s Exact tests were followed up with the z-test of two proportions with Bonferroni correction or multiple Fisher’s Exact tests with Bonferroni correction for multiple comparisons.

To evaluate functional connectivity, we investigated both within- and between-network connectivity. For both measures of functional connectivity, the following group comparisons were investigated: (i) all mutation carriers versus control subjects; (ii) presymptomatic mutation carriers versus control subjects; and (iii) symptomatic mutation carriers versus control subjects. We did not compare presymptomatic and symptomatic mutation carriers directly. Inherent to the disease, there is a considerable age gap between both groups (mean age difference: 23 years) which cannot be adjusted for without losing a significant amount of power.

#### 2.4.1 Within-network connectivity

Functional connectivity within the RSNs was investigated both on a whole network level and on a voxel-based level, to not only assess effects across whole networks, but also investigate potential regional effects. Whole network mean functional connectivity scores (*z* scores) were extracted for each subject. For each network, a binary mask of the RSN template (first thresholded at *z* = 3, similar to Smith et al., 2009) was overlaid on top of the subject’s spatial map created by the dual regression to select only voxels located within the RSN. Mean functional connectivity within the network was then calculated by averaging the *z* values of the selected voxels. In order to analyze only functional tissue and not tissue destroyed by hemorrhages (i.e. microbleeds and/or intracerebral hemorrhages), our approach was twofold. First, gray matter (GM) volume was included as a covariate in the analyses to adjust for differences in network volume between subjects. Second, as large destructive hemorrhages may have an impact on functional connectivity beyond what can be corrected for by gray matter volume corrections, we adopted a more rigorous analysis approach by excluding networks containing gross ICH damage from analysis completely.

First, group differences in mean functional connectivity between mutation carriers and control subjects were assessed using ANCOVAs including gray matter volume correction. For each of the nine RSNs we performed an ANCOVA with age, sex and GM volume of the given RSN added as covariates, with subsequent Bonferroni correction for multiple comparisons, accepting statistical significance at *p* < .0056 (0.05/9). We chose to correct for GM volume on a network level, instead of a whole brain GM volume correction, to account for local (per RSN) rather than global atrophy. GM volumes per network were calculated by overlaying a binary mask of the RSN on top of each subject’s GM partial volume map to extract only the volume of gray matter belonging to the network, and corrected for head size. Additionally, to examine the influence of symptomatology (presymptomatic or symptomatic) and identify if changes in functional connectivity can already be seen in the presymptomatic stage of the disease, we performed nine ANCOVAs with group (control, presymptomatic, symptomatic) as the fixed factor and age, sex and GM volume of the given RSN added as covariates, accepting statistical significance at *p* < .0056. Post hoc analyses with Bonferroni adjustment were used to assess differences in functional connectivity between presymptomatic mutation carriers and control subjects, and symptomatic mutation carriers and control subjects.

Next, we excluded all networks with functional tissue loss due to ICHs from analysis. For each subject, presence of intracerebral hemorrhages in each of the nine networks was determined by visual inspection of susceptibility weighted images overlaid with RSN templates. Per network, an ANCOVA was performed including only participants without major ICH damage in the given network. Group (control, presymptomatic, symptomatic) was the fixed factor and age, sex, and GM volume of the RSN were added as covariates. Post hoc analyses with Bonferroni adjustment were performed, accepting statistical significance at *p* < .0056.

Furthermore, to examine potential regional effects, rather than whole network effects, we studied voxel-level group differences within each RSN. To assess group differences between mutation carriers and control subjects, a two-sample *t*-test was performed using a general linear model (GLM) in FSL’s randomise (Winkler, Ridgway, Webster, Smith, & Nichols, 2014) with 5000 permutations. In the GLM, age and sex were added as covariates (demeaned by subtracting the overall mean from each individual score), as well as a voxelwise confound covariate of GM volume. To construct the voxelwise confound covariate, we used the ‘feat_gm_prepare’ script of FSL. In this script, subjects’ GM partial volume maps are smoothed to match resting state images, registered to standard space and demeaned to be suitable as a covariate in the GLM. The resulting statistical maps of randomise were Family-Wise Error (FWE) corrected for multiple comparisons by the Threshold-Free Cluster Enhancement technique (TFCE; Smith & Nichols, 2009), accepting statistical significance at FWE-corrected *p* < .05. Two additional two-sample *t*-tests were performed in the same way, comparing presymptomatic- and symptomatic mutation carriers separately against the control subjects.

#### 2.4.2 Between-network connectivity

Between-network connectivity was assessed using the FSLNets package (http://fsl.fmrib.ox.ac.uk/fsl/fslwiki/FSLNets). Subject-specific time courses from the dual regression were used to calculate partial correlations (Fisher’s *r*-to-*z* transformed) between all nine RSNs. Two-sample *t*-tests were performed using FSL’s randomise with 5000 permutations and age, sex and whole brain GM volume added as covariates to assess group differences (FWE-corrected *p* < .05). We compared all mutation carriers against control subjects, as well as presymptomatic- and symptomatic mutation carriers separately against the control subjects. To further explore between-network connectivity, we additionally calculated connectivity between subsystems of networks, rather than between networks as a whole, using a higher decomposition of the RSNs (*n* = 42), see Supplementary Materials.

#### 2.4.3 Association with lesion burden

Further, to assess if there was an association between lesion burden and functional connectivity, we performed linear regression analyses in SPSS. The composite score of total MRI burden of SVD in CAA was entered into the model as the independent variable, whole network mean functional connectivity scores of each RSN were entered as dependent variables, and age, sex and GM volume per RSN were added as covariates.

## 3 Results

Demographics and characteristics of the study sample are shown in Table 1. Mutation carriers had significantly more ICHs, lobar CMBs, WMHs, CSO-EPVS, higher occurrence of cSS and higher scores of total MRI burden of SVD in CAA. No differences were found for age, sex, risk factors, MMSE score, and whole brain gray matter volume. Presymptomatic mutation carriers did not differ significantly from control subjects, whereas symptomatic mutation carriers were older, had a higher incidence of hyperlipidemia, lower MMSE scores, lower whole brain gray matter volume and significantly higher incidence of MRI markers of the disease than control subjects.

**Table 1:** Demographics and characteristics of participants.

		Mutation carriers					
	Control subjects (*n* = 29)	All (*n* = 24)	*p*	Presymptomatic (*n* = 11)	*p*	Symptomatic (*n* = 13)	*p*
Age (years)	45 [20 – 67]	50.5 [20 – 63]	0.694	32 [20 – 51]	0.089	55 [45 – 63]	**0.032**
Sex			0.728		0.715		1.000
Male	11 (37.9%)	8 (33.3%)	–	3 (27.3%)	–	5 (38.5%)	–
Female	18 (62.1%)	16 (66.7%)	–	8 (72.7%)	–	8 (61.5%)	–
Hypertension	6 (20.7%)	6 (25%)	0.709	0 (0%)	0.162	6 (46.2%)	0.141
Diabetes	0 (0%)	2 (8.3%)	0.200	1 (9.1%)	0.275	1 (7.7%)	0.310
Cardiovascular disease	0 (0%)	1 (4.2%)	0.453	0 (0%)	–	1 (7.7%)	0.310
Hyperlipidemia	2 (6.9%)	5 (20.8%)	0.224	0 (0%)	1.000	5 (38.5%)	**0.021**
MMSE score	29 [27 – 30]	29.5 [22 – 30]	0.551	30 [29 – 30]	0.112	29 [22 – 30]	**0.016**
Whole brain GM volume (cm^3^)	664.5 (58.6)	642.3 (78.2)	0.247	703.3 (56.1)	0.166	586.4 (47.3)	**< 0.001**
ICHs present	0 (0%)	13 (54.2%)	**< 0.001**	1 (9.1%)	0.275	12 (92.3%)	**< 0.001**
Median no. of ICHs	0 [0 – 0]	6 [0 – 41]	**< 0.001**	0 [0 – 8]	1.000	15 [0 – 41]	**< 0.001**
Lobar CMBs present	2 (6.9%)	15 (62.5%)	**< 0.001**	2 (18.2%)	0.300	13 (100%)	**< 0.001**
Median no. of lobar CMBs	0 [0 – 1]	14.5 [0 – 468]	**< 0.001**	0 [0 – 32]	1.000	48 [2 – 468]	**< 0.001**
WMHs volume (cm^3^)	1.7 [0.2 – 14.1]	12.3 [1.1 – 82.4]	**< 0.001**	3.4 [1.1 – 21.4]	0.725	47.3 [10.8 – 82.4]	**< 0.001**
WMHs rating			**< 0.001**		0.056		**< 0.001**
pvWMH Fazekas ≤ 2 and dWMH Fazekas ≤ 1	28 (96.6%)	8 (33.8%)	**–**	8 (72.7%)	–	0 (0%)	–
pvWMH Fazekas 3 and/or dWMH Fazekas ≥ 2	1 (3.4%)	16 (66.7%)	–	3 (27.3%)	–	13 (100%)	–
CSO-EPVS			**< 0.001**		0.254		**< 0.001**
Degree 0–2	22 (75.9%)	6 (25.0%)	–	6 (54.5%)	–	0 (0%)	**–**
Degree 3–4	7 (24.1%)	18 (75.0%)	–	5 (45.5%)	–	13 (100%)	**–**
cSS			**< 0.001**		0.275		**< 0.001**
Focal	0 (0%)	4 (16.7%)	–	0 (0%)	–	4 (30.8%)	–
Disseminated	0 (0%)	9 (37.5%)	–	1 (9.1%)	–	8 (61.5%)	–
Composite score of total MRI burden of SVD in CAA	0 [0 – 2]	5 [0 – 6]	**< 0.001**	0 [0 – 6]	0.146	6 [3 – 6]	**< 0.001**

*Note*. Data are mean (*SD*), number (%), or median [range]. MMSE = Mini-Mental State Examination. GM = Gray matter. ICHs = intracerebral hemorrhages. CMBs = cerebral microbleeds. WMHs = white matter hyperintensities. pvWMH = periventricular WMH. dWMH = deep WMH. CSO-EVPS = centrum semiovale enlarged perivascular spaces. cSS = cortical superficial siderosis. Reported *p*-values are from statistical (post hoc) tests versus control subjects with bold-face indicating significant differences (after Bonferroni correction, where applicable).

### 3.1 Within-network connectivity

**Fig. 1:**
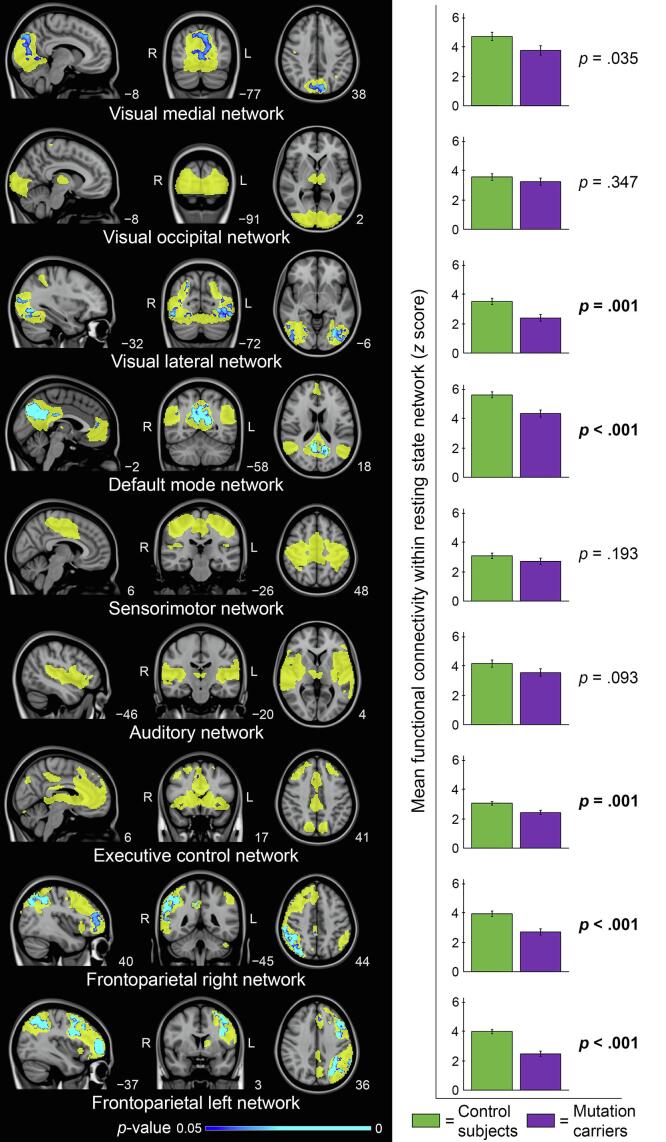
Group differences of within-network connectivity for mutation carriers versus control subjects. Left panel: for each resting state network (yellow) significant voxel-level group differences (TFCE-corrected) are displayed in blue, indicating a reduction in connectivity, overlaid on the MNI standard anatomical image. The most informative sagittal, coronal, and axial slices per network are displayed. Right panel: mean whole network functional connectivity (*z* scores) of each network for control subjects (green) and mutation carriers (purple). Means adjusted for age, sex, and gray matter volume per network with standard error bars are displayed. Bold-faced *p*-values indicate significant differences between groups after Bonferroni correction (*p* < .0056). (For interpretation of the references to color in this figure legend, the reader is referred to the web version of this article.)

Group differences of within-network connectivity for mutation carriers versus control subjects are shown in Fig. 1. In the right panel of Fig. 1, bar graphs show mean functional connectivity within each network (*z* scores, adjusted for age, sex and GM volume of the given RSN) for control subjects and mutation carriers. For all networks, mutation carriers (purple) showed lower functional connectivity within the networks, compared with control subjects (green). The difference is significant for the visual lateral, default mode, executive control, frontoparietal right, and frontoparietal left networks, after Bonferroni correction (*p*-values ≤ 0.001). Exact values of adjusted means and standard errors are provided in Supplementary Table 1. In the left panel of Fig. 1, voxel-level group differences (FWE-corrected) are displayed in blue, overlaid on top of the network (yellow) and the MNI standard space image. Mutation carriers showed lower functional connectivity for parts of the visual medial, visual lateral, default mode, executive control, frontoparietal right, and frontoparietal left networks. MNI coordinates and associated brain regions of local maxima of the significant clusters of voxels are provided in Supplementary Table 2.

**Fig. 2:**
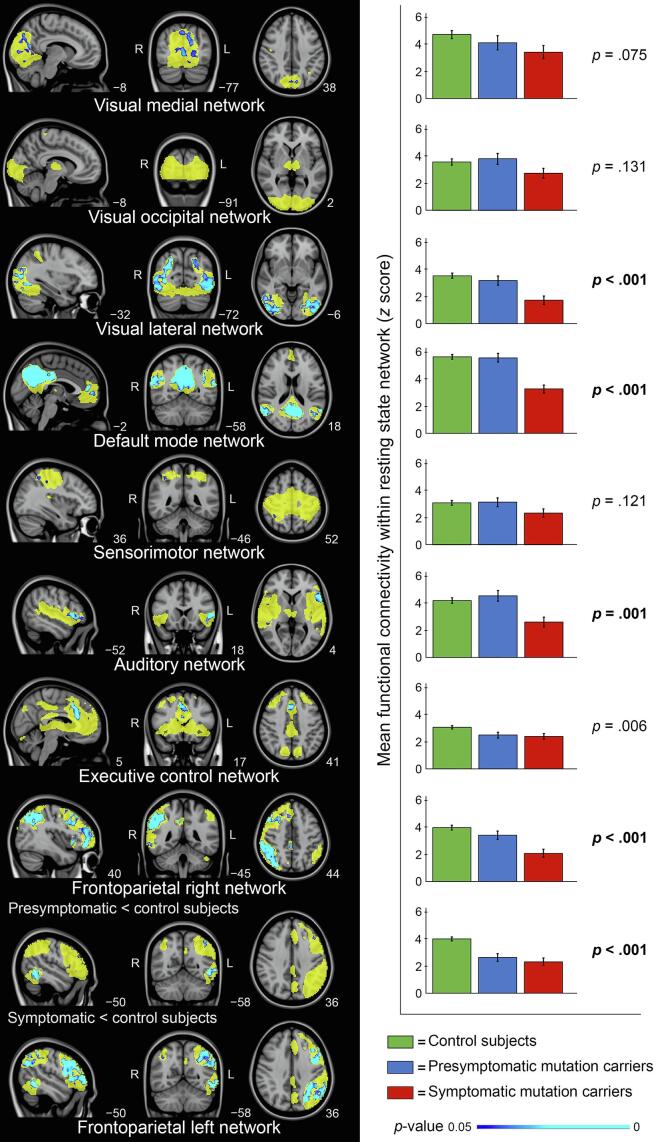
Group differences of within-network connectivity for presymptomatic- and symptomatic mutation carriers versus control subjects. Left panel: for each resting state network (yellow) significant voxel-level group differences (TFCE-corrected) are displayed in blue, indicating a reduction in connectivity, overlaid on the MNI standard anatomical image. The contrast shown for each network is *symptomatic mutation carriers < controls*, unless otherwise stated. The most informative sagittal, coronal, and axial slices per network are displayed. Right panel: mean whole network functional connectivity (*z* scores) of each network for control subjects (green), presymptomatic mutation carriers (blue), and symptomatic mutation carriers (red). Means adjusted for age, sex, and gray matter volume per network with standard error bars are displayed. Presented *p*-values are from ANCOVAs, pairwise comparisons *p*-values can be found in Supplementary Table 3. Bold-faced *p*-values indicate significant differences between the three groups after Bonferroni correction (*p* < .0056). (For interpretation of the references to color in this figure legend, the reader is referred to the web version of this article.)

Group differences of within-network connectivity for presymptomatic- and symptomatic mutation carriers versus control subjects are shown in Fig. 2. Similar to Fig. 1, mean functional connectivity within each network is shown in bar graphs in the right panel of Fig. 2, where mutation carriers are now separated in presymptomatic- (blue) and symptomatic mutation carriers (red). Significant differences were present between the three groups for the visual lateral (*F*(2, 47) = 11.70, *p* < .001), default mode (*F*(2, 47) = 24.79, *p* < .001), auditory (*F*(2, 47) = 7.56, *p* = .001), frontoparietal right (*F*(2, 47) = 14.62, *p* < .001), and frontoparietal left (*F*(2, 47) = 21.72, *p* < .001) networks, after Bonferroni correction (*p* < .0056). Compared to control subjects, functional connectivity was significantly lower in symptomatic mutation carriers for all of the aforementioned networks (0.001 < p ≤ 0.002), whereas in presymptomatic mutation carriers only the frontoparietal left network had significantly lower functional connectivity (*p* < .001). Adjusted means, standard errors, and *p*-values of pairwise comparisons between presymptomatic- and symptomatic mutation carriers versus control subjects are reported in Supplementary Table 3.

The left panel of Fig. 2 shows that symptomatic mutation carriers demonstrated regionally decreased functional connectivity (blue) within the visual medial, visual lateral, default mode, sensorimotor, auditory, executive control, frontoparietal right, and frontoparietal left networks. For the frontoparietal left network, voxel-level decreased functional connectivity was also found for the presymptomatic mutation carriers versus control subjects. MNI coordinates and associated brain regions of the local maxima of the significant clusters of voxels are provided in Supplementary Table 4.

For the analyses that included only networks without major ICH damage, all networks were included for control subjects and presymptomatic mutation carriers, whereas 69 out of 117 (13 subjects × 9 RSNs) networks were included for symptomatic mutation carriers. For each network, number of participants included, adjusted means, standard errors, and *p*-values of pairwise comparisons between presymptomatic- and symptomatic mutation carriers versus control subjects are reported in Supplementary Table 5. Significant differences were found between the three groups for the default mode (*F*(2, 44) = 18.97, *p* < .001), auditory (*F*(2, 45) = 6.07, *p* = .005), frontoparietal right (*F*(2, 41) = 7.53, *p* = .002), and frontoparietal left (*F*(2, 44) = 19.20, *p* < .001) networks, after Bonferroni correction (*p* < .0056). Compared to control subjects, functional connectivity was significantly lower in the frontoparietal left network for both presymptomatic mutation carriers (*n* = 11, *p* < .001), and symptomatic mutation carriers (*n* = 10, *p* < .001). Additionally, symptomatic mutation carriers had significantly lower functional connectivity in the default mode network (*n* = 10, *p* < .001), and frontoparietal right network (*n* = 7, *p* = .002).

### 3.2 Between-network connectivity

**Fig. 3:**
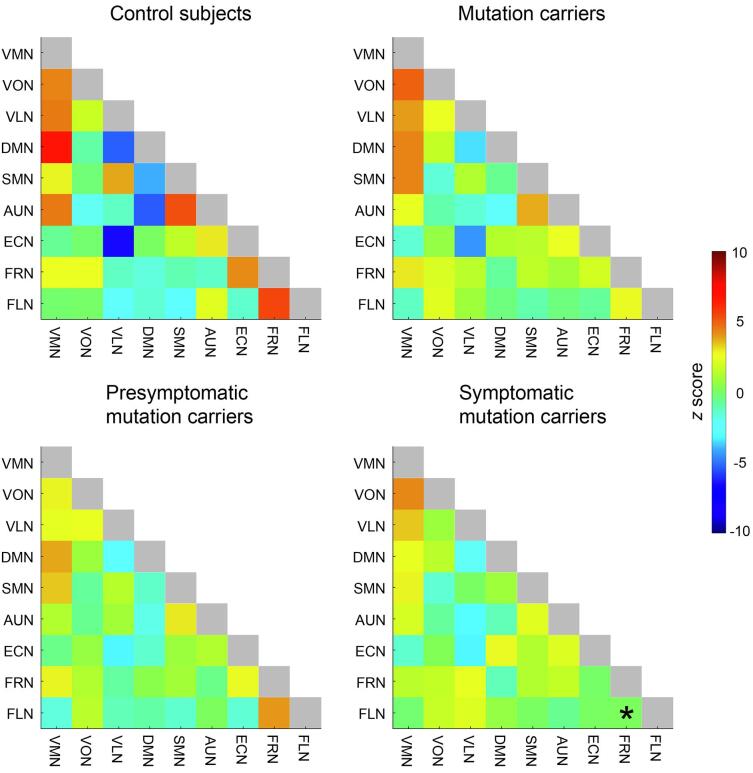
Partial correlation matrices of between-network connectivity for control subjects (top left), all mutation carriers (top right), presymptomatic mutation carriers (bottom left) and symptomatic mutation carriers (bottom right). Correlations have been transformed to *z* scores using Fisher’s *r*-to-*z*-transformation. VMN = visual medial network. VON = visual occipital network. VLN = visual lateral network. DMN = default mode network. SMN = sensorimotor network. AUN = auditory network. ECN = executive control network. FRN = frontoparietal right network. FLN = frontoparietal left network. The asterisk indicates a significant difference with control subjects after correcting for multiple comparisons.

Partial correlations (*r*-to-*z* transformed) between each pair of resting state networks are shown in a matrix per group in Fig. 3. Yellow-to-red colors indicate a positive correlation between networks, for example between the default mode network and visual medial network in control subjects (*z* = 6.81), whereas light-blue-to-dark-blue colors indicate an inverse correlation between networks, for example between the executive control network and visual lateral network in control subjects (*z* =  − 6.51). Visually, the matrices of all mutation carriers, and presymptomatic- and symptomatic mutation carriers separately, tend to be more in the middle of the color spectrum (greener) than the matrix of control subjects, indicating a loss of connectivity strength. No significant differences were found after correcting for multiple comparisons between the matrices of all mutation carriers, and presymptomatic mutation carriers versus control subjects. For the symptomatic mutation carriers, connectivity between the frontoparietal right and frontoparietal left network was significantly lower than in control subjects (*z* = 5.49 for controls subjects versus *z* = 0.11 for symptomatic mutation carriers, *p* = .019). The high-dimensionality analysis assessing connectivity between 42 subcomponents of the RSNs showed similar results, with a visual loss of connectivity (green) in the matrices of all mutation carriers, and presymptomatic- and symptomatic mutation carriers separately. Two partial correlations were significantly different in mutation carriers versus control subjects, involving subcomponents of the default mode, auditory and frontoparietal networks, but not in presymptomatic- or symptomatic carriers separately; see Supplementary Materials for more details.

### 3.3 Association with lesion burden

Linear regression analyses between lesion burden and functional connectivity were performed in the mutation carriers for each network. Due to multicollinearity between symptomatology, age and total SVD score in CAA (lesion burden), we were unable to obtain reliable results. Presymptomatic carriers are younger and have relatively low total SVD in CAA scores compared to symptomatic carriers who are older and score high on the lesion burden scale (Pearson correlation between age and total SVD score: *r* = 0.90). We chose not to remove age from the model as lower functional connectivity is related to aging (Ferreira & Busatto, 2013) and this would lead to omitted variable bias, resulting in an overestimation of the coefficient estimates.

Next, we performed regression analysis in the subgroups (presymptomatic and symptomatic) separately, but again multicollinearity and limited variability of the total SVD score within each subgroups rendered the regression models unstable and unreliable. Thus, we were unable to determine whether lesion load is associated with functional connectivity.

## 4 Discussion

In conclusion, our data show that functional connectivity is decreased in D-CAA mutation carriers across the brain compared to age-matched control subjects. Some degradation can already be identified in the presymptomatic stage of the disease, but deterioration of functional connectivity is most pronounced in symptomatic mutation carriers.

Our main finding is that functional brain connectivity is globally attenuated in D-CAA patients. Since D-CAA can be considered a ‘clean’ model for CAA and given the predilection of CAA pathology to the posterior brain regions, particularly the occipital cortex (Freeze et al., 2019, Kövari et al., 2013, Vinters and Gilbert, 1983), we expected to find significant differences between groups for resting state networks encompassing the occipital cortex. Remarkably, we found reduced functional connectivity in almost all networks for mutation carriers. Not only were networks affected that encompassed posterior brain regions, but also frontally involved networks such as the default mode, executive control and frontoparietal networks, showed diminished functional connectivity in mutation carriers. Our finding that in CAA functional connectivity is affected in the entire brain and not particularly the occipital lobe, is partly consistent with structural brain connectivity studies, where structural brain connectivity is decreased in widespread areas of the brain rather than focused in the occipital cortex (Reijmer et al., 2015, Schouten et al., 2019). Taken together, our data contribute to the idea that CAA-related brain damage extends beyond the areas of most severe pathology and is a global, rather than a focal, phenomenon.

We found that aberrant functional connectivity can already be identified in the presymptomatic stage of the disease. Attenuated functional connectivity was observed in the frontoparietal left network in presymptomatic mutation carriers compared with control subjects. Regional functional connectivity changes within the network were found in temporooccipital, parietal, and frontal regions (i.e. middle frontal gyrus and frontal pole), indicating that frontal brain regions are affected early in the disease. In CAA, a damage trajectory from posterior to anterior brain regions related to disease severity has been demonstrated (Kövari et al., 2013, Reijmer et al., 2016, Vinters and Gilbert, 1983). For example, Reijmer and colleagues (2016) found that in patients with moderate CAA, structural brain connectivity was affected in posterior brain regions, whereas in more severe cases of CAA damage to structural connectivity extended to frontal brain regions. Our results indicate that the functional architecture of the brain is affected early on in the disease in a spatially distributed manner. We did not observe increased functional connectivity in any of the networks in presymptomatic mutation carriers. In some neurodegenerative diseases increased functional connectivity is found in the preclinical stage of the disease which is sometimes interpreted as a functional compensation mechanism of the brain to preserve cognitive function (Dennis and Thompson, 2014, Zhou et al., 2017). For instance, increased functional connectivity has been identified in cognitively normal Apolipoprotein E allele e4 carriers, a genetic risk factor for AD, which is interpreted as a reallocation of resources to be able to maintain proper cognitive function (Filippini et al., 2009, Ye et al., 2017). It could also be hypothesized that there may be compensatory mechanisms at play in presymptomatic CAA patients, considering they are still cognitively normal (van Rooden et al., 2016). However, we did not observe evidence for compensatory mechanisms in presymptomatic CAA patients.

Decreased functional connectivity was most pronounced in symptomatic mutation carriers. This, combined with the finding that decreased functional connectivity was limited in presymptomatic mutation carriers, supports the view that CAA progresses at a nonlinear rate, which accelerates when lesion load increases (Reijmer et al., 2016). Most symptomatic mutation carriers (12/13) presented with intracerebral hemorrhages, but we did not find evidence suggesting ICHs influenced the functional connectivity measures. The spatial distribution of ICHs was heterogeneous among participants, minimizing the influence on the group as a whole, and the distribution of ICHs did not match the distribution of functional connectivity differences found. For example, the parietal cortex was least affected by ICHs (9% of all ICHs) in our study sample, yet we found major functional connectivity differences in the parietal cortex. More importantly, results were significant after a gray matter volume correction was applied and analyses excluding networks with gross ICH damage produced similar results to analyses including all networks. The visual lateral network and the auditory network did not reach significance in the analyses excluding ICH damaged networks, whereas significant differences were found in the analyses including all networks. However, the auditory network just missed the adjusted threshold of *p* < .0056 with a *p*-value of 0.0060, and the visual lateral network only included three symptomatic mutation carriers, substantially lowering the power of the analysis.

Both within- and between-network connectivity are important to understand brain function as information not only flows within segregated systems (i.e. networks), but also between systems for information integration. Changes in between-network connectivity that can be related to brain function (e.g. cognition) have been observed in non-demented elderly (Chan et al., 2014, Geerligs et al., 2015, Varangis et al., 2019, Zonneveld et al., 2019) as well as in preclinical up to advanced dementia patients (Brier et al., 2012, Dennis and Thompson, 2014, Wang et al., 2015). In fact, between-network connectivity was identified as one of the most powerful predictors to classify individual subjects with Alzheimer’s disease (de Vos et al., 2018). Distinct patterns of between-network connectivity degradation have been identified between non-demented individuals and AD patients (Chhatwal et al., 2018), further demonstrating the relative importance of between-network correlations in understanding disease mechanisms. Even though we could not identify significant between-network connectivity differences for most internetwork correlations – most likely because of a lack of statistical power due to limited sample size and strict multiple comparisons corrections – a trend of connectivity loss can be observed. Most between-network correlations regressed to zero, indicating that the functional organization of the brain is degrading in CAA patients.

Overall, functional connectivity is most affected in symptomatic mutation carriers and it appears symptomatic patients are the driving force behind findings in all mutation carriers grouped together versus control subjects. It should be noted we should be cautious directly comparing findings from the different analyses. Power of the analyses is different and effects of covariate corrections might also impact results differentially within subgroups. This might explain why we identified significantly lower functional connectivity in the executive network in mutation carriers versus control subjects, but not in any of the subgroups separately. Subgroup analyses have lower power due to a reduced sample size, but may be more sensitive than analyses in all mutation carriers because of reduced variability within each subgroup. For example, in symptomatic carriers, significantly lower connectivity was found in the auditory network, which was not significant in all mutation carriers. Partial volume corrections for gray matter volume likely has a greater influence on the analysis in symptomatic mutation carriers, as there is more gray matter loss in this group compared to the other groups. As a consequence, there is less network volume in symptomatic carriers which could have impacted the functional connectivity measures and comparison with control subjects. However, we are confident that functional connectivity differences found do not merely reflect GM volume effects as findings from analysis excluding networks with major ICH, i.e. excluding networks with extreme volume reductions, were similar to results including all networks. Different GM volume corrections were used between analyses to attempt to be as precise as possible within each analysis. Additionally, we attempted to gain as much insight into functional connectivity differences by investigating connectivity at multiple levels: more sensitive to local effects through voxel-level within-network analysis and between-network connectivity of RSN subcomponents, and more general effects through mean whole within-network connectivity scores and connectivity between whole RSNs. Considering different power and sensitivity effects, we must be cautious directly comparing findings from the different analyses and groups.

The resting state networks have been associated with numerous behavioral and cognitive functions (Laird et al., 2011, Smith et al., 2009). Brain connectivity – both structural and functional – may be an important mechanism through which widespread lesions in CAA result in behavioral outcome. In CAA, aberrant structural brain connectivity patterns have been related to cognitive impairment (Reijmer et al., 2015). From a cognitive network neuroscience perspective, cognition is supported and facilitated through (functional) networks (Medaglia et al., 2015, Mišić and Sporns, 2016). Interestingly, the profile of cognitive impairment in CAA (i.e. impairments in global cognition, processing speed, executive function, language, and memory; Boyle et al., 2015, Case et al., 2016, van Rooden et al., 2016) corresponds with functions associated with the networks that show a loss of within-network functional connectivity in (mostly symptomatic) mutation carriers. In presymptomatic carriers, attenuated functional connectivity in the frontoparietal left network might be hypothesized to be an early marker of cognitive deterioration. The frontoparietal networks are associated with global cognitive ability and cognitive control (Marek & Dosenbach, 2018), yet no cognitive deterioration has been found in our sample of presymptomatic mutation carriers (van Rooden et al., 2016). As network connectivity is thought to underlie cognition (Medaglia et al., 2015, Mišić and Sporns, 2016), it could be hypothesized that lower connectivity in the frontoparietal network might be a first sign of cognitive degeneration not captured by cognitive behavioral tests. Unfortunately, it is not possible to directly investigate the potential relationship between functional connectivity and brain function in this study. We cannot investigate the link with cognitive decline in presymptomatic mutation carriers due to a lack of cognitive spread in our sample of presymptomatic carriers (see van Rooden et al., 2016). Cognitive deficits were identified in our group of symptomatic mutation carriers (see van Rooden et al., 2016), however the study is underpowered to compare two experimental measures in only 13 symptomatic patients, substantially inflating the risk of false positive findings.

An intrinsic limitation of functional connectivity analysis concerns the blood-oxygen-level-dependent (BOLD) signal underlying functional MRI acquisition. Functional connectivity findings should be interpreted with caution, considering the implications of the BOLD signal. Functional connectivity is mostly interpreted as a measure of neuronal activity, however, the BOLD signal is a very rich signal containing both neural and vascular components. The vascular component of the BOLD signal is often overlooked in the interpretation of functional connectivity findings (Archila-Meléndez et al., 2020). It is possible that the lower functional connectivity observed in this study might be related to differences in the vascular component of the BOLD signal, rather than differences in neuronal activity. Alterations in neurovascular coupling can affect temporal correlations between brain regions, thereby increasing or decreasing the measured functional connectivity (Archila-Meléndez et al., 2020, Liu, 2013). Impaired vascular reactivity has been identified in the occipital cortex of sporadic and both presymptomatic and symptomatic hereditary CAA patients (Dumas et al., 2012, Peca et al., 2013, Switzer et al., 2016, van Opstal et al., 2017), but not in the motor cortices (Peca et al., 2013). As it is not yet clear to what extent stimulation induced BOLD signals can be compared to resting state BOLD signals (Liu, 2013), we cannot be sure of the exact underlying process that resulted in decreased functional connectivity in mutation carriers. Nevertheless, our findings of altered BOLD connectivity throughout the brain show that functional connectivity is a very interesting and potentially sensitive marker of brain damage in CAA.

An important limitation of the study is the relatively small sample size, which reduces our statistical power, especially when comparing presymptomatic- and symptomatic mutation carriers separately to control subjects. However, hereditary CAA is a rare disease, which makes it difficult to collect larger samples. The great benefit of D-CAA as a model for CAA is that it allowed us to investigate CAA without confounding factors, such as AD pathology and other age-related comorbidities, and therefore study the independent effect of CAA. As this is the first study to investigate functional brain connectivity in CAA specifically, further research is needed to see if our findings can be replicated and translated to sporadic CAA.

To summarize, our data show that the intrinsic functional organization of the brain is damaged in patients with Dutch CAA, even before overt symptoms of the disease are present. CAA affects network connectivity independent of aging and other neurodegenerative processes. Given the high prevalence of CAA and its comorbid nature, our findings indicate that when brain connectivity is investigated in elderly subjects or subjects with Alzheimer’s disease and/or dementia, the presence and severity of CAA should be accounted for. Impaired functional connectivity is mainly related to symptomatic CAA and can therefore be considered to be a late consequence of the disease.

## 5 Declarations of interest

N.D., J.v.d.G., S.A.R.B.R., M.A.v.B., M.J.H.W., J.P.C., M.E.G., S.M.G., and S.v.R. declare that they have no known competing financial interests or personal relationships that could have appeared to influence the work reported in this paper. G.M.T. reports consultancy support from Novartis, Lilly, Teva, and independent support from Dutch Organization for Scientific Research, and the Dutch Heart & Brain Foundations.

## Funding

This work was supported by the National Institutes of Health [R01NS070834]. The funding source had no involvement in study design, collection, analysis and interpretation of data, writing of the report, or in the decision to submit the article for publication.

## CRediT authorship contribution statement

**Nadieh Drenth:** Conceptualization, Methodology, Software, Formal analysis, Investigation, Data curation, Writing - original draft, Writing - review & editing, Visualization. **Jeroen Grond:** Conceptualization, Methodology, Validation, Writing - original draft, Writing - review & editing, Visualization, Supervision, Project administration. **Serge A.R.B. Rombouts:** Methodology, Validation, Writing - review & editing, Supervision. **Mark A. Buchem:** Conceptualization, Resources, Writing - review & editing. **Gisela M. Terwindt:** Conceptualization, Investigation, Resources, Data curation, Writing - review & editing. **Marieke J.H. Wermer:** Conceptualization, Investigation, Resources, Data curation, Writing - review & editing. **Jasmeer P. Chhatwal:** Validation, Writing - review & editing. **M. Edip Gurol:** Validation, Writing - review & editing. **Steven M. Greenberg:** Conceptualization, Validation, Resources, Data curation, Writing - review & editing, Funding acquisition. **Sanneke Rooden:** Conceptualization, Methodology, Investigation, Resources, Data curation, Writing - original draft, Writing - review & editing, Supervision, Project administration.

## Declaration of Competing Interest

The authors declare that they have no known competing financial interests or personal relationships that could have appeared to influence the work reported in this paper.
