# Supplementary material for: Cerebral amyloid angiopathy is associated with decreased functional brain connectivity
Source: Neuroimage Clin. 2020 Dec 24;29:102546. doi: 10.1016/j.nicl.2020.102546 (PMC7806879; doi:10.1016/j.nicl.2020.102546)
Supplement: Supplementary data 1 [file mmc1.pdf]

**SUPPLEMENTARY MATERIALS**

# Cerebral amyloid angiopathy is associated with decreased functional brain connectivity

Nadieh Drenth[a], Jeroen van der Grond[a], Serge A.R.B. Rombouts[a,b,c], Mark A. van Buchem[a], Gisela M. Terwindt[d], Marieke J.H. Wermer[d], Jasmeer P. Chhatwal[e], M. Edip Gurol[e], Steven M. Greenberg[e], Sanneke van Rooden[a]

[a] Department of Radiology, Leiden University Medical Center, Leiden, The Netherlands.

[b] Leiden University, Institute of Psychology, Leiden, The Netherlands.

[c] Leiden Institute for Brain and Cognition, Leiden, The Netherlands.

[d] Department of Neurology, Leiden University Medical Center, Leiden, The Netherlands

[e] Department of Neurology, Massachusetts General Hospital, Harvard Medical School, Boston, USA

# Supplementary Methods

## High-dimensionality between-network connectivity analysis

In addition to the large-scale resting state network (RSN) templates, Smith et al. (2009) further decomposed the networks through a high-dimensionality independent component analysis restrained at 70 components. To examine connectivity between subcomponents of networks, rather than whole RSNs, the 70 templates together with a CSF and WM template were entered into a dual regression. Of the 70 components, 45 were classified as nonartifactual (Smith et al., 2009). Similar to the large-scale network analyses, three components corresponding to the cerebellar network were excluded, resulting in 42 components included in the analysis. Subject-specific time courses from the dual regression were used to calculate partial correlations (Fisher's *r*-to-*z* transformed) between the 42 components in FSLNets (http://fsl.fmrib.ox.ac.uk/fsl/fslwiki/FSLNets). Two-sample *t*-tests were performed using FSL's randomise with 5000 permutations and age, sex and whole brain GM volume added as covariates to assess group differences (FWE-corrected $p < .05$). We compared all mutation carriers against control subjects, as well as presymptomatic- and symptomatic mutation carriers separately against control subjects.

## Supplementary Results

Supplementary Table 1

*Mean functional connectivity within each resting state network (z scores) for control subjects and mutation carriers*

| | Control subjects | | Mutation carriers | | | |
|---|---|---|---|---|---|---|
| RSN | *M* (*SD*) | $M_{adj}$ (*SE*) | *M* (*SD*) | $M_{adj}$ (*SE*) | % difference | *p* |
| Visual medial | 4.75 (2.04) | 4.71 (0.29) | 3.70 (1.97) | 3.75 (0.32) | −20.4% | .035 |
| Visual occipital | 3.60 (1.42) | 3.56 (0.22) | 3.20 (1.74) | 3.25 (0.24) | −8.7% | .347 |
| Visual lateral | 3.57 (1.17) | 3.52 (0.21) | 2.34 (1.75) | 2.40 (0.23) | −31.8% | **.001** |
| Default mode | 5.69 (0.90) | 5.62 (0.21) | 4.27 (2.16) | 4.35 (0.23) | −22.6% | **< .001** |
| Sensorimotor | 3.11 (1.04) | 3.08 (0.19) | 2.67 (1.28) | 2.71 (0.21) | −12.0% | .193 |
| Auditory | 4.18 (1.27) | 4.16 (0.24) | 3.51 (1.93) | 3.55 (0.26) | −14.7% | .093 |
| Executive control | 3.07 (0.74) | 3.05 (0.12) | 2.42 (0.84) | 2.43 (0.14) | −20.3% | **.001** |
| Frontoparietal right | 3.98 (1.05) | 3.95 (0.19) | 2.68 (1.25) | 2.71 (0.21) | −31.4% | **< .001** |
| Frontoparietal left | 4.02 (1.05) | 3.99 (0.15) | 2.44 (1.05) | 2.48 (0.17) | −37.8% | **< .001** |

*Note*. RSN = resting state network. $M_{adj}$ = mean adjusted for age, sex and gray matter volume per resting state network. Percentage difference reflects the difference between the adjusted means. Bold-faced *p*-values indicate significant differences between groups after Bonferroni correction ($p < .0056$).

Supplementary Table 2

*MNI coordinates and associated brain regions of local maxima of within-network clusters that showed significantly decreased functional connectivity ($p_{FWE}$ < .05) for mutation carriers compared with control subjects. Results are corrected for age, sex and gray matter volume per voxel*

| | | | Peak voxel MNI coordinates | | | |
|---|---|---|---|---|---|---|
| RSN | No. of voxels | $p_{\text{FWE}}$ | X | Y | Z | Peak voxel location[a] |
| **Mutation carriers < control subjects** | | | | | | |
| Visual medial | 1640 | .014 | −12 | −84 | 40 | Superior lateral occipital cortex |
| Visual lateral | 2731 | .006 | 54 | −56 | −20 | Temporooccipital inferior temporal gyrus |
| | 1902 | .008 | −30 | −76 | −6 | Occipital fusiform gyrus |
| | 14 | .045 | 36 | −40 | 42 | Posterior supramarginal gyrus |
| Default mode | 3331 | < .001 | 6 | −52 | 22 | Posterior cingulate gyrus |
| | 169 | .008 | 44 | −68 | 38 | Superior lateral occipital cortex |
| | 54 | .024 | −2 | 44 | 10 | Anterior cingulate gyrus |
| Executive control | 15 | .036 | 6 | 16 | 42 | Paracingulate gyrus |
| Frontoparietal right | 2416 | < .001 | 16 | −74 | 52 | Superior lateral occipital cortex |
| | 468 | .011 | 38 | 46 | 12 | Frontal pole |
| | 157 | .018 | 48 | 16 | 40 | Middle frontal gyrus |
| | 152 | .004 | 10 | −44 | 34 | Posterior cingulate gyrus |
| | 98 | .011 | 28 | 16 | 54 | Middle frontal gyrus |
| | 51 | .024 | 60 | −50 | −6 | Temporooccipital middle temporal gyrus |
| Frontoparietal left | 4048 | < .001 | −40 | 36 | −16 | Frontal orbital cortex |

Supplementary Table 2 (continued).

| RSN | No. of voxels | $p_{FWE}$ | Peak voxel MNI coordinates X | Y | Z | Peak voxel location[a] |
|---|---|---|---|---|---|---|
| Frontoparietal left | 2194 | < .001 | −26 | −68 | 30 | Superior lateral occipital cortex |
| | 1077 | < .001 | −54 | −62 | −18 | Temporooccipital inferior temporal gyrus |
| | 116 | .006 | −4 | 14 | 52 | Paracingulate gyrus |
| | 92 | .006 | 40 | −52 | 52 | Angular gyrus |
| | 51 | .018 | −4 | −40 | 30 | Posterior cingulate gyrus |

*Note.* RSN = resting state network.

[a] Identified with the Harvard-Oxford Cortical Structural Atlas.

Supplementary Table 3

*Mean functional connectivity within each resting state network (z scores) for presymptomatic- and symptomatic mutation carriers versus control subjects*

| | Control subjects | | Presymptomatic mutation carriers | | | | Symptomatic mutation carriers | | | |
|---|---|---|---|---|---|---|---|---|---|---|
| RSN | *M* (*SD*) | $M_{adj}$ (*SE*) | *M* (*SD*) | $M_{adj}$ (*SE*) | % difference | *p* | *M* (*SD*) | $M_{adj}$ (*SE*) | % difference | *p* |
| Visual medial | 4.75 (2.04) | 4.72 (0.29) | 5.08 (2.08) | 4.11 (0.52) | −12.9% | .912 | 2.53 (0.80) | 3.42 (0.49) | −27.5% | .095 |
| Visual occipital | 3.60 (1.42) | 3.57 (0.22) | 4.51 (1.72) | 3.80 (0.39) | 6.4% | 1.000 | 2.08 (0.64) | 2.75 (0.36) | −23.0% | .182 |
| Visual lateral | 3.57 (1.17) | 3.53 (0.19) | 3.75 (1.68) | 3.17 (0.34) | −10.2% | 1.000 | 1.15 (0.42) | 1.72 (0.32) | −51.3% | **< .001** |
| Default mode | 5.69 (0.90) | 5.64 (0.17) | 6.10 (1.62) | 5.57 (0.31) | −1.2% | 1.000 | 2.72 (1.03) | 3.27 (0.29) | −42.0% | **< .001** |
| Sensorimotor | 3.11 (1.04) | 3.09 (0.18) | 3.52 (1.31) | 3.13 (0.33) | 1.3% | 1.000 | 1.96 (0.71) | 2.34 (0.31) | −24.3% | .131 |
| Auditory | 4.18 (1.27) | 4.19 (0.21) | 5.11 (1.55) | 4.54 (0.38) | 8.4% | 1.000 | 2.16 (0.91) | 2.61 (0.36) | −37.7% | **.002** |
| Executive control | 3.07 (0.74) | 3.05 (0.12) | 2.83 (0.84) | 2.49 (0.22) | −18.4% | .087 | 2.07 (0.69) | 2.39 (0.21) | −21.6% | .026 |
| Frontoparietal right | 3.98 (1.05) | 3.97 (0.18) | 3.67 (1.00) | 3.41 (0.32) | −14.1% | .382 | 1.84 (0.71) | 2.08 (0.30) | −47.6% | **< .001** |
| Frontoparietal left | 4.02 (1.05) | 3.99 (0.15) | 3.15 (1.06) | 2.64 (0.28) | −33.8% | **< .001** | 1.84 (0.59) | 2.32 (0.26) | −41.9% | **< .001** |

*Note.* RSN = resting state network. $M_{adj}$ = mean adjusted for age, sex and gray matter volume per resting state network. Percentage difference reflects the difference between the adjusted means of the group versus control subjects. Reported *p*-values are from pairwise comparisons versus control subjects, where bold-face indicates significant differences between groups after Bonferroni correction ($p < .0056$).

Supplementary Table 4

*MNI coordinates and associated brain regions of local maxima of within-network clusters that showed significantly decreased functional connectivity ($p_{FWE}$ < .05) for presymptomatic mutation carriers and symptomatic mutation carriers compared with control subjects. Results are corrected for age, sex and gray matter volume per voxel*

| | | | Peak voxel MNI coordinates | | | |
|---|---|---|---|---|---|---|
| RSN | No. of voxels | $p_{\text{FWE}}$ | X | Y | Z | Peak voxel location[a] |
| **Presymptomatic mutation carriers < control subjects** | | | | | | |
| Frontoparietal left | 703 | .001 | −48 | −56 | −8 | Temporooccipital inferior temporal gyrus |
| | 176 | .009 | −44 | 24 | 40 | Middle frontal gyrus |
| | 131 | .016 | −42 | −62 | 50 | Superior lateral occipital cortex |
| | 42 | .027 | −34 | 58 | 2 | Frontal pole |
| | 33 | .029 | −42 | 42 | −12 | Frontal pole |
| | 15 | .044 | −32 | 52 | 18 | Frontal pole |
| **Symptomatic mutation carriers < control subjects** | | | | | | |
| Visual medial | 1819 | .012 | −18 | −68 | 16 | Supracalcarine cortex |
| | 23 | .047 | −12 | −76 | −16 | Lingual gyrus |
| Visual lateral | 4902 | .001 | 30 | −80 | 38 | Superior lateral occipital cortex |
| | 2650 | .004 | −52 | −74 | −14 | Inferior lateral occipital cortex |
| Default mode | 5674 | < .001 | 8 | −48 | 6 | Posterior cingulate gyrus |
| | 1153 | .001 | 62 | −56 | 18 | Angular gyrus |
| | 913 | .002 | −38 | −72 | 38 | Superior lateral occipital cortex |
| | 351 | .005 | −2 | 44 | 10 | Anterior cingulate gyrus |

Supplementary Table 4 (continued).

| RSN | No. of voxels | $p_{FWE}$ | Peak voxel MNI coordinates X | Y | Z | Peak voxel location[a] |
|---|---|---|---|---|---|---|
| Sensorimotor | 76 | .031 | 34 | −48 | 52 | Superior parietal lobule |
| Auditory | 427 | .004 | −58 | 14 | 4 | Inferior frontal gyrus, pars opercularis |
| | 50 | .024 | 50 | −38 | 2 | Temporooccipital middle temporal gyrus |
| | 31 | .030 | 56 | 20 | 2 | Inferior frontal gyrus, pars triangularis |
| | 16 | .028 | −64 | −48 | 8 | Temporooccipital middle temporal gyrus |
| | 11 | .041 | −58 | −60 | 8 | Temporooccipital middle temporal gyrus |
| Executive control | 750 | .002 | 6 | 26 | 24 | Anterior cingulate gyrus |
| | 45 | .022 | 0 | 44 | 16 | Paracingulate gyrus |
| | 14 | .029 | −26 | 42 | 4 | Frontal pole |
| Frontoparietal right | 3189 | .001 | 64 | −46 | 28 | Angular gyrus |
| | 2189 | .001 | 46 | 22 | 44 | Middle frontal gyrus |
| | 292 | .015 | 60 | −38 | −10 | Posterior middle temporal gyrus |
| | 184 | .008 | 42 | 16 | −4 | Insular cortex |
| | 174 | .004 | 10 | −44 | 32 | Posterior cingulate gyrus |
| | 43 | .024 | 8 | 26 | 44 | Paracingulate gyrus |
| Frontoparietal left | 4020 | .001 | −40 | 44 | 2 | Frontal pole |
| | 2500 | < .001 | −24 | −68 | 36 | Superior lateral occipital cortex |
| | 598 | .001 | −54 | −56 | −12 | Temporooccipital inferior temporal gyrus |
| | 108 | .020 | −2 | −74 | 44 | Precuneous cortex |
| | 79 | .014 | 42 | −62 | 46 | Superior lateral occipital cortex |

*Note.* RSN = resting state network.

[a] Identified with the Harvard-Oxford Cortical Structural Atlas.

Supplementary Table 5

*Mean functional connectivity within each resting state network (z scores) for presymptomatic- and symptomatic mutation carriers versus control subjects including only networks free of major ICH damage*

| | Control subjects ($n$ = 29) | | Presymptomatic mutation carriers ($n$ = 11) | | | | Symptomatic mutation carriers | | | | |
|---|---|---|---|---|---|---|---|---|---|---|---|
| RSN | $M$ ($SD$) | $M_{adj}$ ($SE$) | $M$ ($SD$) | $M_{adj}$ ($SE$) | % difference | $p$ | $n$ | $M$ ($SD$) | $M_{adj}$ ($SE$) | % difference | $p$ |
| Visual medial | 4.75 (2.04) | 4.88 (0.31) | 5.08 (2.08) | 4.26 (0.54) | -12.7% | 1.000 | 6 | 2.85 (1.02) | 3.74 (0.74) | -23.4% | .490 |
| Visual occipital | 3.60 (1.42) | 3.74 (0.24) | 4.51 (1.72) | 3.95 (0.41) | 5.6% | 1.000 | 3 | 2.59 (0.74) | 3.36 (0.80) | -10.2% | 1.000 |
| Visual lateral | 3.57 (1.17) | 3.65 (0.21) | 3.75 (1.68) | 3.28 (0.36) | -10.1% | 1.000 | 3 | 1.32 (0.54) | 2.28 (0.69) | -37.5% | .198 |
| Default mode | 5.69 (0.90) | 5.71 (0.17) | 6.10 (1.62) | 5.62 (0.31) | -1.6% | 1.000 | 10 | 2.92 (1.06) | 3.40 (0.33) | -40.5% | **< .001** |
| Sensorimotor | 3.11 (1.04) | 3.11 (0.19) | 3.52 (1.31) | 3.17 (0.33) | 1.9% | 1.000 | 10 | 1.75 (0.60) | 2.15 (0.35) | -30.9% | .058 |
| Auditory | 4.18 (1.27) | 4.20 (0.21) | 5.11 (1.55) | 4.54 (0.38) | 8.1% | 1.000 | 11 | 2.20 (0.97) | 2.72 (0.39) | -35.2% | .006 |
| Executive control | 3.07 (0.74) | 3.08 (0.13) | 2.83 (0.84) | 2.52 (0.22) | -18.2% | .098 | 9 | 2.13 (0.75) | 2.45 (0.26) | -20.5% | .112 |
| Frontoparietal right | 3.98 (1.05) | 4.00 (0.19) | 3.67 (1.00) | 3.46 (0.33) | -13.5% | .483 | 7 | 2.10 (0.81) | 2.33 (0.41) | -41.8% | **.002** |
| Frontoparietal left | 4.02 (1.05) | 4.02 (0.16) | 3.15 (1.06) | 2.67 (0.28) | -33.6% | **< .001** | 10 | 1.79 (0.64) | 2.31 (0.30) | -42.5% | **< .001** |

*Note.* RSN = resting state network. $M_{adj}$ = mean adjusted for age, sex and gray matter volume per resting state network. Percentage difference reflects the difference between the adjusted means of the group versus control subjects. Reported $p$-values are from pairwise comparisons versus control subjects, where bold-face indicates significant differences between groups after Bonferroni correction ($p < .0056$).

**High-dimensionality between-network connectivity**

Partial correlations ($r$-to-$z$ transformed) between each of the 42 components are shown in a matrix per group in the top panel of Supplementary Figure 1, where yellow-to-red colors indicate a positive correlation and light-blue-to-dark-blue indicate an inverse correlation between components. In general, connectivity matrices of all mutation carriers, presymptomatic- and symptomatic mutation carriers are visually more green than control subjects, indicating an overall loss of connectivity (Supplementary Figure 1). Two partial correlations between RSN subcomponents were significantly different between mutation carriers and control subjects (indicated by black boxes and highlighted for visualization purposes in Supplementary Figure 1). The bottom panel of Supplementary Figure 1 displays the spatial maps of the components for which significant group differences were identified. Brain regions and standard RSNs associated with the components are listed in Supplementary Table 6. Connectivity between components 14 (related to auditory network) and 29 (related to frontoparietal left network) was positive in control subjects ($z = 2.55$), but negative in all mutation carriers ($z = -3.31$, $p = .008$). Connectivity between components 21 (related to frontoparietal left and right networks) and 32 (related to DMN) was less negative in mutation carriers ($z = -2.74$) than control subjects ($z = -6.63$, $p = .030$). We only identified significant differences with control subjects for the mutation carriers grouped together, but not in the subgroups (i.e. presymptomatic- or symptomatic carriers) separately, which might simply reflect a lack of power in the subgroups.

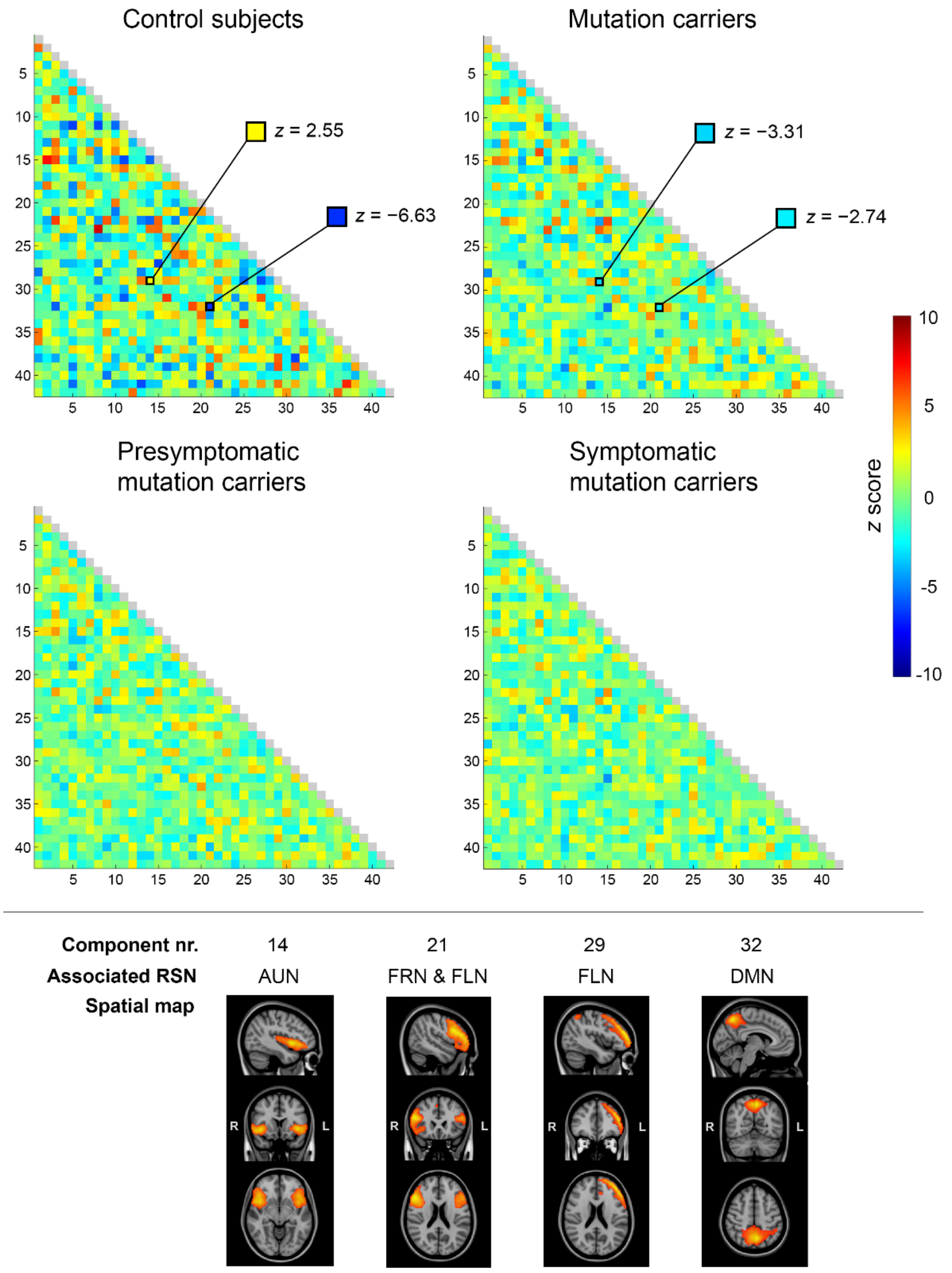


*Supplementary Figure 1.* Top panel: partial correlation matrices of connectivity between all components for control subjects (top left), all mutation carriers (top right), presymptomatic mutation carriers (bottom left) and symptomatic mutation carriers (bottom right). Correlations have been transformed to *z* scores using Fisher's *r*-to-*z*-transformation. Black borders indicate significant

differences ($p_{FWE} < .05$) in correlation strength between groups. Bottom panel: components for which significant group differences were identified. The most informative sagittal, coronal, and axial slices per component are displayed overlaid on the MNI standard anatomical image. RSN = resting state network. AUN = auditory network. FRN = frontoparietal right network. FLN = frontoparietal left network. DMN = default mode network.

Supplementary Table 6

*Associated standard resting state networks and brain regions of components that showed significant group differences in partial correlations between the components.*

| Component nr. | Associated RSN | Associated brain regions |
|---|---|---|
| 14 | Auditory | Bilateral insula extending to the frontal orbital cortex |
| 21 | Frontoparietal right & Frontoparietal left | Mainly corresponds to the frontoparietal right network, comprising the right middle frontal gyrus, precentral gyrus, inferior frontal gyri pars opercularis and pars triangularis, frontal pole, extending to the frontal orbital cortex and insular cortex. To the left a smaller, but similarly located cluster is limited to the inferior frontal gyri pars triangularis and pars opercularis corresponding to the frontoparietal left network. |
| 29 | Frontoparietal left | A large lateral cluster in the frontal lobe comprising the frontal pole, middle frontal gyrus, inferior frontal gyrus pars opercularis, precentral gyrus, and superior frontal gyrus. Smaller clusters are located in the frontal lobe at the anterior cingulate gyrus and paracingulate gyrus and in the parietal lobe at the angular gyrus, and posterior and anterior supramarginal gyri |
| 32 | Default mode | Bilateral precuneous extending to superior parietal lobule and superior lateral occipital cortex. Smaller clusters are located in the bilateral superior frontal and middle frontal gyri. |

*Note.* RSN = resting state network.
